# Wheeled Mobility

**DOI:** 10.1155/2015/138176

**Published:** 2015-04-01

**Authors:** Alicia M. Koontz, Dan Ding, Yih-Kuen Jan, Sonja de Groot, Andrew Hansen

**Affiliations:** ^1^Human Engineering Research Laboratories, VA Pittsburgh Healthcare System, Pittsburgh, PA 15206, USA; ^2^Department of Rehabilitation Science and Technology, University of Pittsburgh, Pittsburgh, PA 15260, USA; ^3^Department of Kinesiology and Community Health, University of Illinois at Urbana-Champaign, Champaign, IL 61820, USA; ^4^Amsterdam Rehabilitation Research Center, Reade, 1040 HG Amsterdam, The Netherlands; ^5^University of Groningen, University Medical Center Groningen, Center for Human Movement Sciences, 9700 AB Groningen, The Netherlands; ^6^Rehabilitation Engineering Research Program, Minneapolis VA Healthcare System, Minneapolis, MN 55417, USA; ^7^Department of Physical Medicine and Rehabilitation, Program in Rehabilitation Science, University of Minnesota, Minneapolis, MN 55455, USA

Independence in mobility is one of the most important determinants of quality of life for individuals with disabilities [1]. While independent mobility is achievable with a wide variety of mobility-related technologies in existence today (e.g., prosthetic devices, powered orthotics, exoskeletons, etc.), wheeled mobility devices continue to make up the greatest portion of assistive devices in use. There are currently about 2.7 million wheelchair users in the United States (US) [2]. The powered and manual mobility market globally (with US being the largest regional market) is projected to grow exponentially due to aging baby boomers and increasing longevity [3]. This anticipated increased growth in the numbers of wheelchair users and that many wheelchair users continue to develop secondary disabilities along with experiencing other complications and barriers associated with wheelchair use brought us to this special issue.

This special issue features seven original research studies and seven development studies that advance our knowledge about secondary disabilities associated wheelchair use, measurement and training tools, and new assistive devices and adaptations. One ongoing thread of research in our field is to better understand how certain wheelchair features and tasks can lead to disabling secondary conditions such as pressure ulcers and upper limb pain. Y.-S. Yang et al. give us insight into injury risks associated with wheelchair standing by quantifying the amount of shear displacement that occurs between the body and backrest and seat, the ranges of motion of the lower limb joints, and the forces that act on the knee and foot during the seat-to-stand transition. Y.-S. Lin et al. show us that performing wheelchair push-ups for pressure relief reduces the shoulder joint's subacromial space and may predispose wheelchair users to shoulder impingement. This study also found that greater narrowing of the space after performing a repetitive rotator cuff task to simulate overuse was associated with increased years of wheelchair use and higher levels of shoulder pain. B. Slavens et al. describe in detail the propulsion techniques used by children and young adult manual wheelchair users with spinal cord injury. They found that the weight-normalized forces and moments these children and youth used to propel were similar to those reported in adult samples and, thus, identified children and youth as targets for interventions that help to prevent upper limb pain and overuse injuries. M. M. B. Morrow et al. in their comprehensive and detailed magnetic resonance imaging analysis of wheelchair users with shoulder pain tell us where the most common and exact locations of shoulder tendon tears are and describe other widespread abnormalities lending to a deeper understanding into patterns of pathological shoulder findings among manual wheelchair users.

Other original research studies in this issue extend our understanding of the demands of manual wheelchair use and address either the personal or wheelchair attributes that affect the amount of effort required. D. Gagnon et al. show that pushing up a ramp increases shoulder mechanical and muscular effort and that success with steeper slopes is likely influenced by one's strength generating capacity and body mass index. A. Mandy et al. demonstrate that grip forces are reduced with a one-arm drive system, a type of adaptation often prescribed to individuals with hemiplegia, that “ties” the rear wheels together and can be driven and steered with one arm and foot compared to a similar type of system that does not “tie” the rear wheels together. F. Chenier and R. Aissaoui give us promising data on the use of carbon fibre in wheelchair frame construction in reducing the mechanical work load required to travel a certain distance while at the same time reducing the whole-body vibrations transmitted to the body from the ground.

The development of new robotic assistive devices and training programs and tools to enhance mobility and outcome measurement are additional areas of recent interest and growth. G. G. Grindle et al. describe a new wheelchair mounted robot arm called “StrongArm” capable of moving a person from their wheelchair to another surface and back eliminating the heavy lifting that would normally be required from a caregiver. E. M. Giesbrecht et al. detail the development and components of a new wheelchair skills home-based program tailored specifically for the needs of older adults. D. C. Kamaraj et al. establish the content validity of two new clinical tools, one specifically designed for screening for the appropriateness of power mobility and another for driving assessment. C.-Y. Tsai et al. find that wheelchair users who independently transfer and score higher (e.g., better) on the transfer assessment instrument, a new clinical scale used to assess a patient's transfer technique, exhibit the type of biomechanics that may protect them from developing upper limb pain and injuries.

The ongoing development, evaluation, or refinement of more accurate monitoring devices and measurement tools is important for quantifying the user performance and the interface between the user and the wheelchair. M. Ojeda and D. Ding discover that wearing a triaxial accelerometer around the upper arm combined with a wheel-mounted data logging system is a viable option to collect temporal data such as stroke frequencies and the cumulative exposure to forces on the upper limbs that occurs from daily wheelchair use. B. Mason et al. find that while using a highly portable miniature wheelchair data logger system to collect elite wheelchair activity works well for certain metrics, it falls short of being able to fully describe the intricacies of wheelchair performance on the court when compared to a less portable radiofrequency-based indoor tracking system. C.-W. Lung et al. call to our attention that the peak pressure index metric used to quantify seat interface pressure is sensitive to changes in wheelchair tilt and recline. Their study quantifies the amount of displacement in the metric and offers suggestions about how to remove this effect and allow for more accurate pressure readings.

We hope the science in this special issue will stimulate continued research that shares the same overarching goal of all this work which aims to help wheelchair users to become completely independent and highly functional in their devices, be protected from secondary complications associated with wheelchair use, be fully integrated into society, and be able to live active and healthy lifestyles.
